# Prevalence of *PRKDC* mutations and association with response to immune checkpoint inhibitors in solid tumors

**DOI:** 10.1002/1878-0261.12739

**Published:** 2020-06-30

**Authors:** Yu Chen, Yi Li, Yanfang Guan, Yingying Huang, Jing Lin, Lizhu Chen, Jin Li, Gang Chen, Leong Kin Pan, Xuefeng Xia, Ning Xu, Lianpeng Chang, Zengqing Guo, Jianji Pan, Xin Yi, Chuanben Chen

**Affiliations:** ^1^ Department of Medical Oncology Fujian Medical University Cancer Hospital & Fujian Cancer Hospital Fuzhou China; ^2^ Cancer Bio‐immunotherapy Center Fujian Medical University Cancer Hospital & Fujian Cancer Hospital Fuzhou China; ^3^ Fujian Provincial Key Laboratory of Translational Cancer Medicine Fuzhou China; ^4^ Department of Radiotherapy Fujian Cancer Hospital & Fujian Medical University Cancer Hospital Fuzhou China; ^5^ Geneplus‐Beijing Institute Beijing China; ^6^ Department of Pathology Fujian Medical University Cancer Hospital & Fujian Cancer Hospital Fuzhou China; ^7^ CCIC Group Kuok Kim (Macao) Medical Center III China; ^8^ Hui Xian Medical Center Macao China; ^9^ Department of Urology The First Affiliated Hospital of Fujian Medical University Fuzhou China; ^10^ Department of Radiotherapy Fujian Medical University Cancer Hospital & Fujian Cancer Hospital Fuzhou China

**Keywords:** DNA‐PKcs, immune checkpoint inhibitors, *PRKDC*, tumor microenvironment, tumor mutation burden

## Abstract

Predictive biomarkers of response to immune checkpoint inhibitors (ICI) help to identify cancer patients who will benefit from immunotherapy. Protein kinase, DNA‐activated, catalytic subunit (*PRKDC*) is an important gene for DNA double‐strand break (DSB) repair and central T‐cell tolerance. We aimed to investigate the association between *PRKDC* mutations and tumor mutation burden (TMB), tumor microenvironment (TME), and response to ICI. Whole‐exome sequencing data of 4023 solid tumor samples from the Cancer Genome Atlas (TCGA) and panel‐based sequencing data of 3877 solid tumor samples from Geneplus‐Beijing, China, were used to analyze the TMB. The mRNA expression data of 3541 solid tumor samples from TCGA were used to explore the effect of *PRKDC* mutations on the TME. Four ICI‐treated cohorts were analyzed for verifying the correlation between *PRKDC* mutations and the response to ICI. In both the TCGA and Geneplus datasets, we found that the TMB in *PRKDC* mutation samples was significantly higher than in *PRKDC* wild‐type samples (*P* < 0.05 and *P* < 0.0001, respectively). Further, TCGA datasets showed that *PRKDC* mutation samples were associated with a significantly increased expression of CD8^+^ T cells, NK cells, immune checkpoint, chemokines, etc. compared to *PRKDC* wild‐type samples (*P* < 0.05). In ICI‐treated cohorts, we also found the *PRKDC* mutations were associated with increased survival (median PFS, not reached vs. 6.8 months, HR, 0.2893; 95% CI, 0.1255–0.6672; *P* = 0.0650, Hellmann cohort; median OS, 1184 days vs. 250 days, HR, 0.5126; 95% CI, 0.2715–0.9679; *P* = 0.1020, Allen cohort), and the increase was significant in multivariate analysis (HR, 0.361; 95% CI, 0.155–0.841; *P* = 0.018, Allen cohort; HR, 0.240 95% CI, 0.058–0.998; *P* = 0.050, Hellmann cohort). In summary, we found that *PRKDC* mutation often appeared to co‐exist with deficiency in some other DNA damage repair mechanism and is nonetheless one of the important factors associated with increased TMB, inflamed TME, and better response to ICI.

## Graphical abstract

Protein kinase, DNA‐activated, catalytic subunit (*PRKDC*) is a gene related to DNA damage response (DDR). Here, we found that *PRKDC* mutation often co‐exists with other DNA damage repair deficiencies and is associated with an increased tumor mutation burden, an inflamed tumor microenvironment, and a good response to immune checkpoint inhibitors.

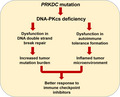

**Abbreviations**
DDR: DNA damage repair
DNA‐PK: DNA‐dependent serine/threonine protein kinase
DNA‐PKcs: DNA‐dependent serine/threonine protein kinase catalytic subunit
DSB: DNA double‐strand break
GSEA: Gene set enrichment analysis
HR: homologous recombination
ICIs: immune checkpoint inhibitors
MMR: mismatch repair
MSI: microsatellite instability
NHEJ: nonhomologous end‐joining
*PRKDC*: protein kinase, DNA‐activated, catalytic subunit
TCGA: The Cancer Genome Atlas
TMB: tumor mutation burden
TME: tumor microenvironment

## 1 Introduction

New immune checkpoint inhibitors (ICI) have changed the therapeutic landscape for many types of cancer [1, 2, 3]. However, only a subset of cancers are responsive to ICI [4]. Several biomarkers, including PD‐L1 expression [5], RNA expression signatures [6], tumor mutational burden (TMB) [7], and lymphocyte infiltration [8], have been reported as possible biomarkers to identify patients who may benefit from ICI.

Gene mutations in the tumor DNA damage repair (DDR) pathway have also been recently reported as an important predictor for the response to ICI therapy. Mutations in DNA polymerase ɛ (*POLE*) and polymerase ƌ (*POLD1*), mismatch repair (MMR) genes, and *BRCA1/BRCA2* reduce genomic stability and can lead to hypermutations [9, 10, 11]. The protein kinase, DNA‐activated, catalytic subunit (*PRKDC*) that encodes the DNA‐dependent serine/threonine protein kinase catalytic subunit (DNA‐PKcs) is a member of the DDR pathway. DNA‐dependent serine/threonine protein kinase (DNA‐PK) is composed of DNA‐PKcs and a heterodimer of Ku proteins (Ku70/Ku80). DNA‐PK is a critical component of the nonhomologous end‐joining (NHEJ) pathway that is mainly involved in DNA double‐strand break (DSB) repair and maintaining genomic integrity [11, 12, 13]. In this study, we performed a comprehensive analysis of *PRKDC* mutations by reviewing the Cancer Genome Atlas (TCGA) database, the Geneplus database, and four available clinical cohorts treated with ICI [7, 14, 15, 16]. Notably, we uncovered that *PRKDC* mutations were significantly associated with a high TMB similar with other DDR pathway‐related gene mutations, including *POLE/D1*, MMR genes, *BRCA1/2*, and the presence of *PRKDC* mutations predicted a superior response to ICI therapy compared with patients with wild‐type *PRKDC*. We highlight the importance of validation of *PRKDC* mutations for the delivery of precise immunotherapy.

## 2 Materials and methods

This study had been informed and approved by the ethics committee of the Fujian Medical University Cancer Hospital. All procedures in the study were conducted conformed to the standards set by the Declaration of Helsinki.

### 2.1 Patients and specimens

From August 12, 2016, to March 4, 2019, 3877 solid tumor samples collected from 3877 patients underwent a panel‐based next‐generation sequencing assays at Geneplus‐Beijing, Beijing, China. The cancer types and number of samples of each cancer type included in Geneplus are displayed in Table S1. All patients signed a written consent. We also analyzed 4023 samples from TCGA (is a cancer research project established by the National Cancer Institute (NCI) and the National Human Genome Research Institute (NHGRI), Bethesda, MD, USA), 110 samples from the Allen cohort [16], 75 samples from the Hellmann cohort [14], 34 samples from the Rizvi cohort [7], and 64 samples from the Snyder cohort [15].

### 2.2 Panel‐based sequencing

#### 2.2.1 Specimen processing and DNA extraction

The genomic DNA from frozen tissue samples was extracted by using the Tissue gDNA exaction Kit (Qiagen, Hilden, Germany). DNA from formalin‐fixed, paraffin‐embedded specimens (FFPE) was isolated by using a commercially available kit (Maxwell® 16 FFPE Plus LEV DNA Purification; Qiagen. catalog: AS1135). The DNA concentration was measured using a Qubit fluorometer and the Qubit dsDNA HS (High Sensitivity) Assay Kit (Invitrogen, Carlsbad, CA, USA).

#### 2.2.2 Library preparation, target capture, and next‐generation sequencing

Sequencing was carried out using Illumina 2 × 75‐bp paired‐end reads on an Illumina HiSeq 3000 instrument according to the manufacturer's recommendations using the KAPA DNA Library Preparation Kit (Kapa Biosystems, Wilmington, MA, USA). Barcoded libraries were hybridized to a customized panel of 1021 genes containing whole exons and selected introns of 288 genes and selected regions of 733 genes, and another panel of 430 genes most frequently mutated in solid tumors. The libraries were sequenced to a uniform median depth (> 500×) and assessed for somatic variants including single nucleotide variants (SNVs), small insertions and deletions (InDels), copy number alterations (CNA), and gene fusions/rearrangements.

#### 2.2.3 Somatic mutation calling

MuTect2 (3.4‐46‐gbc02625) [17] was employed to identify somatic small InDels and SNVs.

### 2.3 Data source

#### 2.3.1 The cancer Genome Atlas dataset

We obtained the WES data of 4023 solid tumors and the mRNA expression data of 3541 solid tumors across 10 tumor types from TCGA. The experimental procedures for DNA and RNA extraction from tumors, library preparation, sequencing, quality control, and subsequent data processing were published previously by TCGA [18]. The mRNA expression was quantified by RSEM (RNA‐seq by expectation‐maximization) [19]. The data were log_2_(*x* + 1)‐transformed before analysis.

#### 2.3.2 Genome and MSI status data in four datasets

Gastric, colorectal, and endometrial tumors are the three types of cancer known to include microsatellite stability (MSS) and microsatellite instability (MSI) subtypes. To explore the association between PRKDC mutations and MSI status and their effects on TMB, we obtained the WES data from 246 endometrial carcinoma, 223 colorectal adenocarcinoma, and 295 gastric adenocarcinoma samples with MSS/MSI subtype information from three studies [20, 21, 22] in TCGA Network, and the whole genome sequencing (WGS) data of 100 gastric adenocarcinoma samples from the Kai Wang cohort [23].

#### 2.3.3 Available clinical cohorts

To further explore the association between *PRKDC* mutations and the clinical benefits of ICI, we analyzed the genomic and clinical data from four clinical cohorts treated with ICI and predicted the neoantigen data from the Hellmann cohort. The first cohort consisted of 75 patients with non‐small‐cell lung cancer (NSCLC) treated with anti‐PD‐1 therapy and anti‐CTLA‐4 therapy (Hellmann cohort) [14]. The second cohort consisted of 110 patients with advanced‐stage melanoma treated with anti‐CTLA‐4 therapy (Allen cohort) [16]. The third cohort was comprised of 64 patients with advanced‐stage melanoma treated with anti‐CTLA‐4 therapy (Snyder cohort) [15]. The last cohort was comprised of 34 patients with NSCLC treated with anti‐PD‐1 therapy (Rizvi cohort) [7].

### 2.4 Biomarker analysis

We defined any nonsynonymous mutations in the gene of interest as ‘mut+’, including missense, nonsense, frameshift indels, in‐frame indels, and splice site mutations.

#### 2.4.1 Tumor mutation burden analysis

Tumor mutation burden was defined as the total somatic nonsynonymous mutation counts in coding regions. TMB was classified into high or low taking the top quartile as the cutoff value.

#### 2.4.2 mRNA expression analysis in immune‐related gene set

The response to ICI has been reported to be related to cytotoxic T cells, NK cells, chemokines, and checkpoints [7, 24]. The immune gene list is based on published articles [25] and [26] that summarized the genes related to CD8 T cells, NK cells, cytotoxic lymphocyte, chemokines, plasmacytoid dendritic cell precursors (pDCs), Th1, macrophages, CD4 T_reg_, CD4 T cells, neutrophils, etc. The mRNA expression of a gene set was defined as the arithmetic mean of transcripts per million values of genes in this gene set.

#### 2.4.3 Gene set enrichment analysis

Gene set enrichment analysis (GSEA) was performed using the javagsea 3.0 Desktop Application (http://software.broadinstitute.org/gsea/index.jsp). The gene sets used for the enrichment analysis were downloaded from the Molecular Signatures Database (MsigDB, http://software.broadinstitute.org/gsea/index.jsp). The gene sets with a false discovery rate (FDR) < 0.05 were considered as significantly enriched. The normalized enrichment score (NES) is the primary statistic for examining gene set enrichment results.

#### 2.4.4 Predicted neoantigen burden

Neoantigens in 75 samples from the Hellmann cohort were estimated [14]. The mutated DNA sequences were virtually translated into corresponding mutated peptide sequences by using Topiary (https://github.com/hammerlab/topiary/) [27]. Topiary was used to run netmhccons (v. 1.1) [28] in order to predict MHC class I binding affinity for all 8–11 mer peptide sequences containing the mutated amino acid. For variants longer than a single residue, all 8–11 mers downstream of the variant were considered. Candidate neoantigens were those peptides with a binding affinity IC_50_ of % 500 nm to one (or more) of the patient‐specific HLA alleles.

### 2.5 Prediction of the functional impact of mutation

We used the functional impact predicting tools SIFT and PolyPhen‐2 HumVar to predict the effects of *PRKDC* mutations on protein function in a clinical case we presented. Mutations with a SIFT score < 0.05 predicted to be deleterious or PolyPhen‐2 HumVar score > 0.5–0.9 were considered possibly damaging and probably damaging (score > 0.9).

### 2.6 Statistical analysis

Statistical analyses were conducted using graphpad prism (version 8.0.1; GraphPad Software, San Diego, CA, USA) and spss version 25.0 (SPSS, Inc., International Business Machines Corporation (IBM), Armonk, NY, USA). If TMB, TNB, and mRNA were normally distributed, a Student *t* test was used to determine the differences between two groups; otherwise, the Mann–Whitney *U* test was used. Logistic regression was used to analyze the influencing factors of TMB‐high. Pearson's correlation was used to analyze the correlation between the length of exons and the mean number of somatic mutations in the exon region for 800 genes with long transcripts including PRKDC, and the fitness lines in scatter plots were plotted with Loess regression which fitted by (weighted) least squares. Kaplan–Meier survival and multivariate Cox regression analyses were used to analyze associations between mutation type and survival, with a *P* value determined by a log‐rank test. HR was determined through Cox regression. The factors associated with survival with *P* < 0.15 in univariable analysis were included in Cox proportion hazard model multivariable analysis. All reported *P* values were two‐tailed, and *P* < 0.05 was considered significant.

## 3 Results

### 3.1 *PRKDC* mutation profile landscape in TCGA and Geneplus

**Fig. 1:**
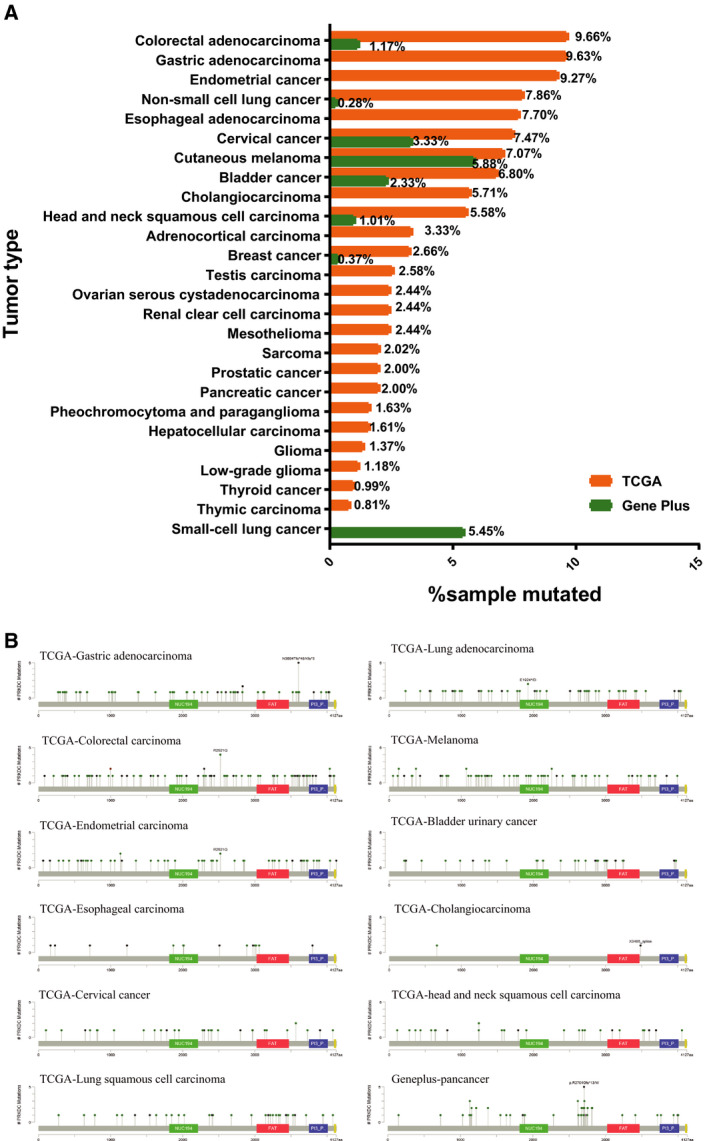
Prevalence of *PRKDC* Mutations. (A) Frequency of *PRKDC* mutations across different tumor types in TCGA and Chinese population (Geneplus). (B) Mutation sites and mutation type of the *PRKDC* gene in TCGA and Geneplus.

We explored the prevalence of *PRKDC* mutations in the TCGA cohort and in the Geneplus cohort (a cohort of a Chinese pan‐cancer population). In the TCGA cohort, colorectal adenocarcinoma had the highest *PRKDC* mutation frequency of 9.66% (51/528), followed by gastric adenocarcinoma with 9.63% (42/436), endometrial cancer 9.27% (23/248), and non‐small‐cell lung cancer 7.86% (81/1031; Fig. 1A) In the Geneplus cohort, melanoma had the highest mutation frequency of 5.88% (1/17), followed by small‐cell lung cancer with 5.45% (3/55) and cervical squamous cell carcinoma 3.33% (1/30; Fig. 1A). *PRKDC* mutation sites were scattered throughout the genes analyzed, and no hotspot mutation was detected in either of the two cohorts (Fig. 1B).

### 3.2 *PRKDC* mutation is associated with increased tumor mutation burden in pan‐cancer

**Fig. 2:**
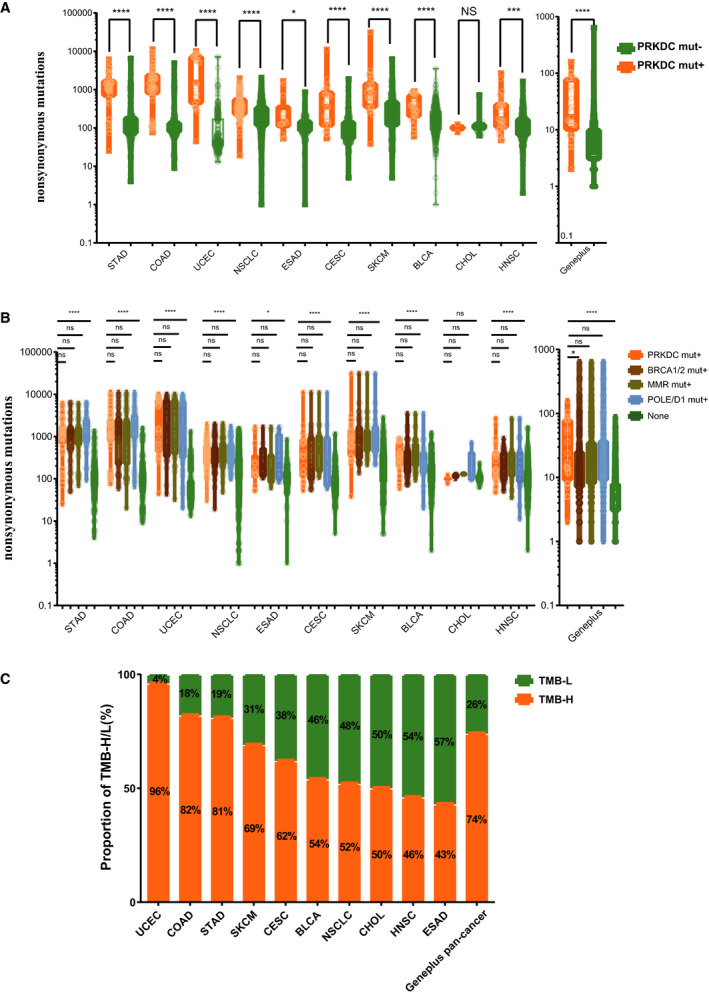
Relationships between TMB and *PRKDC* mutation status. (A) Comparison of TMB between *PRKDC* mutations and *PRKDC* wild‐type samples in TCGA top10 cancers and Geneplus pan‐cancer. TMB, defined as the sum of somatic nonsynonymous mutations. (B) Comparison of TMB between *PRKDC* mutations and other DDR‐gene (including BRCA1/BRCA2, PMS2/MSH2/MSH6/MLH1, POLE/POLD1) mutations in TCGA top10 cancers and Geneplus pan‐cancer. The none group was the referent group, defined as the absence of any of the aforementioned mutations. (C) The proportion of TMB‐high/low status in PRKDC mutation samples in TCGA top10 cancers and Geneplus pan‐cancer. TMB‐H, defined as the upper quartile of tumor all samples' TMB in each cancer type. Statistical significance was calculated using the Mann–Whitney *U* test. ****P < 0.0001; ***P < 0.001; **P < 0.01; *P < 0.05; ns *P *> 0.05. STAD, stomach adenocarcinoma (*N* = 436); COAD, colorectal adenocarcinoma (*N* = 528); UCEC, uterine corpus endometrial carcinoma (*N* = 248); NSCLC, non‐small‐cell lung cancer (*N* = 1031); ESAD, esophageal adenocarcinoma (*N* = 182); CESC, cervical squamous cell carcinoma (*N* = 281); SKCM, human skin cutaneous melanoma (*N* = 368); BLCA, bladder urothelial carcinoma (*N* = 412); CHOL, cholangiocarcinoma (*N* = 35); HNSC, head and neck squamous cell carcinoma (*N* = 502); Geneplus, pan‐cancer samples in Geneplus‐Beijing Institute (*N* =。 3877). (*N*: the number of samples).

To validate the association of *PRKDC* mutations with TMB, we compared the mutation burden of samples with *PRKDC* mutations and samples *PRKDC* wild‐type. In the top 10 cancers with the highest *PRKDC* mutation frequency from the TCGA dataset, the TMB in samples with *PRKDC* mutations was significantly higher than in those without *PRKDC* mutations (median nonsynonymous mutations 1278 vs. 109, *P* < 0.0001 for stomach adenocarcinoma; 1450 vs. 99, *P* < 0.0001 for colorectal adenocarcinoma; 1529 vs. 45, *P* < 0.0001 for uterine corpus endometrioid carcinoma). Other results are shown in (Fig. 2A). Previous studies had shown that mutations in some pivotal DDR pathway genes are associated with genomic instability and increased TMB, including MMR (*PMS2*/*MLH1*/*MSH2*/*MSH6*), *POLE/POLD1,* and *BRCA1/BRCA2* [11]. Our study showed that there was no significant difference in TMB among PRKDC mutation group and MMR gene, POLE/D1, and BRCA1/2 mutation groups. The aforementioned groups all had a higher TMB than the nonmutation group: The nonmutation group was defined as patients without any mutations in the above genes (Fig. 2B). The TMB levels of the top quartile are commonly considered TMB‐high [29], and according to the cutoff value, we found that 96% of PRKDC mutation samples were TMB‐H in the uterine corpus endometrial carcinoma cohort, followed by 82% of the PRKDC mutation samples being TMB‐H in the colorectal adenocarcinoma cohort and 81% in the gastric adenocarcinoma group (Fig. 2C). We then conducted a logistic regression to analyze the influencing factors of TMB‐H. The results showed that the independent risk factors of TMB‐H were PRKDC mutation, BRCA1/2 mutation, POLE/D1 mutation, MMR gene mutation, and age (all *P* < 0.05; Table S2).

Similar results were found in the Geneplus cohort. Patients with *PRKDC* mutations showed significantly higher TMB than those with *PRKDC* wild‐type (median nonsynonymous mutations 17.0 vs. 6.0, *P* < 0.0001). When compared with other DDR genes, the *PRKDC* mutation group had a higher TMB than the *BRCA1/2* mutation group (*P* < 0.05) but was not different from the MMR mutation group or the *POLE/D1* mutation group. The proportion of TMB‐high in the *PRKDC* mutation group was 74% over a pan‐cancer analysis (Fig. 2A–C).

To further explore the association among *PRKDC* mutation, MSI status, and TMB, we included four clinical cohorts covering three cancer types [20, 21, 22]. It showed that 63.89% (46/72) of *PRKDC* mutations subgroups are MSI‐H samples, but *PRKDC* mutation and MSI are not completely overlapped, and the majority of *PRKDC* mutations which did not overlap with MSI‐H group are still TMB‐H (Fig. S1). Among the nonoverlapped 26 *PRKDC* mut+/MSS MSI‐L patients, it also presented a large proportion (20/26) of patients with other DDR genes (BRCA1/2, POLE/D1, or MMR genes) mutation, but four of the rest six *PRKDC* mut+/MSS MSI‐L/other DDR mut− patients were still TMB‐H. Then, we compared the TMB among *PRKDC* mut+/MSI‐H, *PRKDC* mut−/MSI‐H, *PRKDC* mut+/MSS MSI‐L, and *PRKDC* mut−/MSS MSI‐L subgroups in the four cohorts. In the Lander's endometrial carcinoma cohort [20, 21, 22], the TMB of the *PRKDC* mut+ samples is significantly higher than in the *PRKDC* mut− samples in both MSI‐H and MSI‐L/MSS subtypes (median nonsynonymous mutations 558 vs. 220, *P* < 0.001 in MSI‐H subgroup, 5764 vs. 31, *P* < 0.0001 in MSS/MSI‐L subgroup). In the Muzny's colorectal adenocarcinoma cohort [20, 21, 22] and the Vesteinn's gastric adenocarcinoma cohort [20, 21, 22], the TMB of *PRKDC* mut+ samples is significantly higher than in the *PRKDC* mut− samples only in the MSS/MSS‐L subtypes (median nonsynonymous mutations 3209 vs. 69.50, *P* < 0.0001 for colorectal adenocarcinoma, 234 vs. 87, *P* < 0.01 for gastric adenocarcinoma). In the Kai Wang's gastric adenocarcinoma cohort [20, 21, 22], the TMB of *PRKDC* mut+ samples is significantly higher than in the *PRKDC* mut− samples only in the MSI‐H subtypes (median nonsynonymous mutations 1467 vs. 553, *P* < 0.01; Fig. S2). Furthermore, a logistic regression was performed to analyze the influencing factors of TMB‐H in the combined four cohorts. It showed that the independent risk factors of TMB‐H were *PRKDC* mutation, MSI‐H, *BRCA1/2* mutation, MMR gene mutation, and *POLE/D1* mutation (odds ratio, 19.428, 95% CI, 5.525–68.317, *P* = 0.000, *PRKDC* mutation; Table S3).

The *PRKDC* gene has a very long transcript with 12 784 bp, and genes with longer transcripts are generally considered more likely to accumulate somatic mutations by chance. In order to show that the large number of mutations that occur in *PRKDC* is not due to its long transcript, we analyzed whether the length of the transcript was correlated with the average number of somatic mutations in a gene set, which including 800 genes with the length of transcripts more than 8100 bp (8100–104 301 bp). In most of the genes, we found that the length of the transcript correlated with the average number of somatic mutations within them when we analyzed four TCGA patient cohorts (*r* = 0.7537, ****P < 0.0001, Bladder Carcinoma cohort; *r* = 0.7276, ****P < 0.0001, Colorectal Cancer cohort; *r* = 0.7679, ****P < 0.0001, Lung Adenocarcinoma cohort; and *r* = 0.7824, ****P < 0.0001, Head and Neck Squamous Cell Carcinoma cohort; Fig. S3). For example, *TTN*, *SYNE2,* and *RSF1* genes showed a short‐weighted distance from the fitness lines, which indicated according to the trend well. But the *PRKDC* gene was not consistent with the fitness lines (Fig. S3). Thus, the mutations that occur in *PRKDC* are not only due to the longer transcript length.

### 3.3 The correlation of *PRKDC* mutations with signatures of CTL, NK cell infiltration, and inflamed tumor microenvironment

**Fig. 3:**
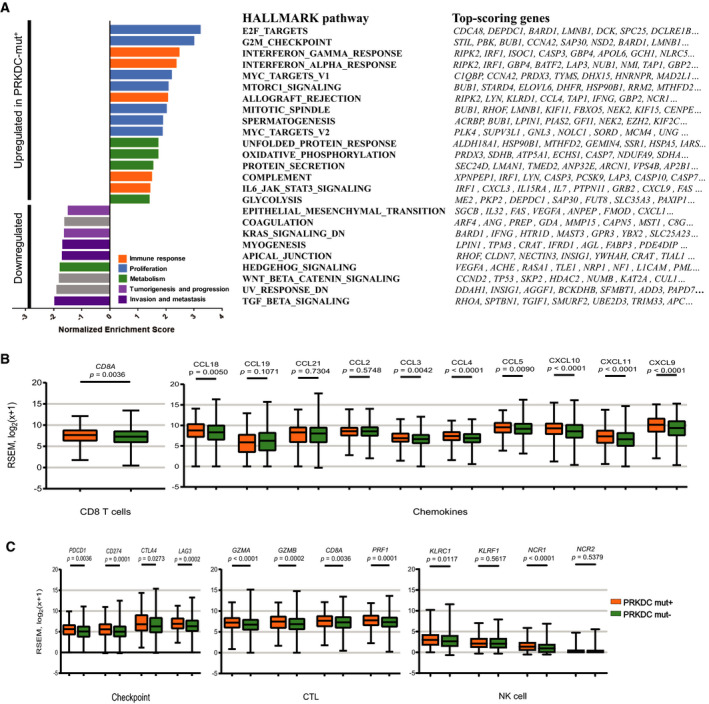
Transcriptome analysis by *PRKDC* mutation status in TCGA top10 cancers. (A) GSEA of hallmark gene sets downloaded from MSigDB database. Hallmark pathways significantly associated with *PRKDC* mutation (FDR. *q* < 0.05; comparing 285 *PRKDC* mutation samples to 3256 *PRKDC* wild‐type samples), and the top 10 genes per set are shown; complete lists are given in Table S1. (B, C) Comparison of the mRNA expression of genes related to immune checkpoints, cytotoxic lymphocyte, NK cells, chemokines, and Th1 cells signature between *PRKDC* mutations and *PRKDC* wild‐type groups in the TCGA top10 cancers analysis. CTL, cytotoxic lymphocyte. Statistical significance was calculated using the Mann–Whitney *U* test in B and C.

To further explore the distinct phenotypic and immunologic states caused by *PRKDC* mutations, we performed GSEA with the Hallmark gene set in the *PRKDC* mutation group and the *PRKDC* wild‐type group based on the TCGA top 10 cancers dataset. Notably, we found that sixteen gene sets were significantly upregulated, including five immune‐related signaling pathways: IFN‐γ response, IFN‐α response, allograft rejection, complement, and IL‐6/JAK/STAT signaling. Nine gene sets were significantly downregulated, including two immune‐related negative signaling pathways (TGF‐β signaling and Wnt/β‐catenin signaling) in the *PRKDC* mutation group (FDR *q* < 0.05; Fig. 3A and Table S4). Previous studies [30] have confirmed that the IFN‐γ pathway is one of the key pathways to induce PD‐L1 expression. TGF‐β plays an important role in promoting tumor immune escape and immunotherapy resistance [24, 31]. The upregulation of the IFN‐γ response and other immune‐related pathways along with downregulation of TGF‐β may contribute to the improvement of ICI therapy in patients with *PRKDC* mutations.

Previous reports showed that the presence of immune cells, especial tumor‐specific T‐cell and NK cell infiltration, which can be estimated using transcriptome signatures, had a significant association with a superior response to ICI [24]. We analyzed 3541 samples of 10 solid tumors from TCGA with a *PRKDC* mutation frequency in the top 10 with both RNA‐seq and WES data. The mRNA expression levels of immune‐related gene clusters were analyzed. Among the 14 selected immune‐related gene clusters (Table S5), the mRNA expression of eight gene clusters was significantly higher in the subgroup with *PRKDC* mutations than in the subgroup having the *PRKDC* wild‐type (*P* < 0.05; Table S6). Specifically, the *PRKDC* mutation subgroup demonstrated dramatically higher levels of mRNA expression than did the *PRKDC* wild‐type subgroup in the following gene clusters: CD8 T cells (CD8A, Fig. 3B), chemokines (CCL18, CCL3, CCL4, CCL5, CXCL10, CXCL11, and CXCL9, Fig. 3B), immune checkpoint (PD‐L1, PD‐1, CTLA‐4, and LAG3, Fig. 3C), cytotoxic T cells (GZMA, GZMB, CD8A, and PRF1, Fig. 3C), and NK cells (KLRC1 and NCR1, Fig. 3C).

### 3.4 *PRKDC* mutations predict favorable clinical benefit to ICI

**Fig. 4:**
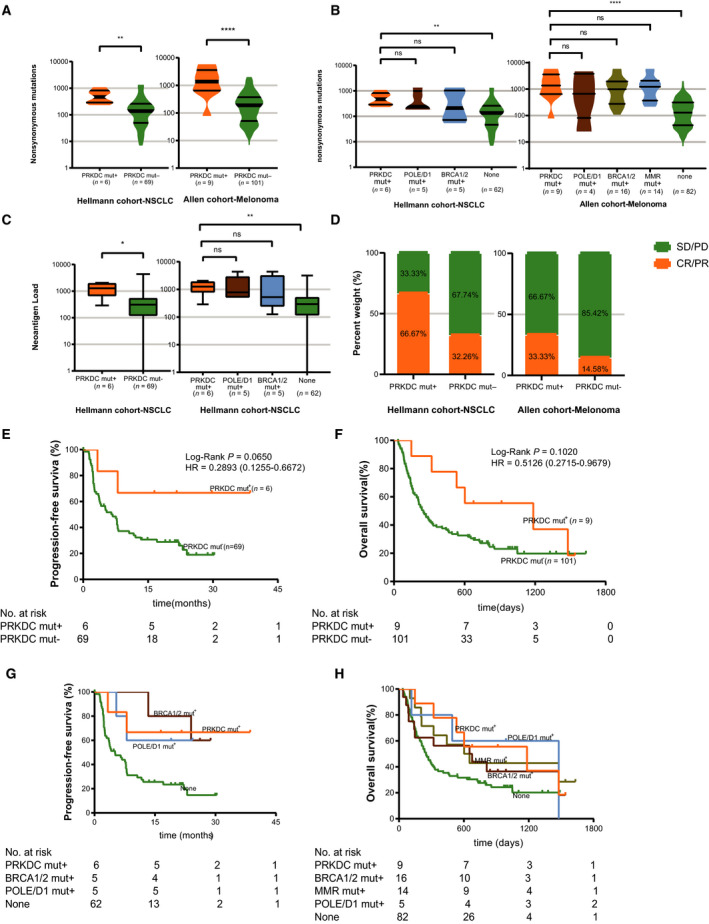
Patients with *PRKDC* mutations showed a favorable clinical benefit from immune checkpoint blockades. (A) Comparison of TMB between *PRKDC* mutations and *PRKDC* wild‐type in two different IO cohorts. (B) Comparison of TMB between *PRKDC* mutation and other DDR‐gene mutations in cohorts. (C) Comparison of neoantigen load between *PRKDC* mutation and *PRKDC* wild‐type in cohorts. (D) Comparison of the ORR between the *PRKDC* mutations and *PRKDC* wild‐type groups from cohorts. (E) Kaplan–Meier survival curves of PFS comparing the *PRKDC* mutations and *PRKDC* wild‐type groups in patients with NSCLC treated with combined PD‐1 and CTLA‐4 blockade from the Hellmann cohort. (F) Kaplan–Meier survival curves of OS comparing the *PRKDC* mutations and *PRKDC* wild‐type groups in patients with melanoma treated with CTLA‐4 blockade from the Allen cohort. (G) Kaplan–Meier survival curves of PFS comparing the *PRKDC* mutations and other DDR‐gene mutation groups in patients with NSCLC treated with combined PD‐1 and CTLA‐4 blockade from the Hellmann cohort. (H) Kaplan–Meier survival curves of OS comparing the *PRKDC* mutations and other DDR‐gene mutation groups in patients with melanoma treated with anti‐CTLA‐4 therapy from the Allen cohort. Statistical significance was calculated using the Mann–Whitney *U* test in comparison of TMB and neoantigen load in A–C. ****P < 0.0001; ***P < 0.001; **P < 0.01; *P < 0.05; ns *P* > 0.05. The *P* value in Kaplan–Meier survival curves was determined by a log‐rank test.

We next examined four independent cohorts to investigate whether patients with *PRKDC* mutations could benefit from ICI therapy. In the first two cohorts [14, 16], a total of 110 patients with melanoma from the Allen cohort who received anti‐CTLA4 therapy and 75 patients with non‐small‐cell lung cancer (NSCLC) from the Hellmann cohort who received combined anti‐PD‐1 and anti‐CTLA4 therapy were analyzed. Patients with *PRKDC* mutations had higher TMB than patients with wild‐type *PRKDC* in both cohorts (Fig. 4A), and the TMB status in the *PRKDC* mutation group was similar to the *BRCA1/2*, *POLE/D1*, and MMR gene mutation groups (Fig. 4B). A higher neoantigen load was found in the *PRKDC* mutation patients in the Hellmann cohort (Fig. 4C).

The Hellmann cohort included 75 patients with NSCLC, including six patients with *PRKDC* mutations for whom progression‐free survival (PFS) was superior to that of *PRKDC* wild‐type patients [median, not reached (NR) vs. 6.8 months; HR, 0.2893; 95% CI, 0.1255–0.6672; *P* = 0.0650, Fig. 4E]. The objective response rates (ORR) of patients with a *PRKDC* mutation and those with a *PRKDC* wild‐type were 66.67% and 32.26%, respectively.

The Allen cohort enrolled 110 patients with metastatic melanoma, and a total of nine patients were identified with *PRKDC* mutations. Compared with the patients with the *PRKDC* wild‐type, the *PRKDC* mutation patients in this cohort also trended toward a longer overall survival (OS; median, 1184 days vs. 250 days; HR, 0.5126; 95% CI, 0.2715–0.9679; *P* = 0.1020, Fig. 4F) and a higher ORR (33.33% vs. 14.58%, Fig. 4D).

We compared the survival of patients with *PRKDC* mutations, MMR gene mutations, *POLE/D1* mutations, and *BRCA1/2* mutations with patients with no mutations in these genes (none mutation subgroup) in the two cohorts. In the Hellmann cohort, we found that *PRKDC* mutations were significantly associated with a longer PFS than in the none mutation subgroup (median, NR vs. 5.092 months; HR, 0.2541; 95% CI, 0.1138–0.5674; *P* = 0.0382, Fig. 4G) and had similar PFS with the *BRCA1/2* mutation and *POLE/D1* mutation subgroups (Fig. 4G). In the Allen cohort, patients with a *PRKDC* mutation showed a trend toward a longer OS than those with no mutation in these DDR genes (median, 1184 days vs. 250 days, HR = 0.5051, 95% CI, 0.2645–0.9646; *P* = 0.0939, Fig. 4H), and also a similar OS to patients with *BRCA1/2* mutations, *POLE/D1* mutations, and MMR gene mutations (Fig. 4H). In multivariate analysis, it showed a significant longer survival in *PRKDC* mutation samples in the two cohorts, after adjustment for clinicopathological characteristics, MMR genes, *POLE/D1*, and *BRCA1/2* (hazard ratio, 0.240 95% CI, 0.058–0.998, *P* = 0.050, Hellmann cohort, hazard ratio, 0.361; 95% CI, 0.155–0.841; *P* = 0.018, Allen cohort; Tables 1 and 2). It suggests that a *PRKDC* mutation is an independent predictive factor of better clinical outcome in ICI‐treated patients.

**Table 1:** Univariable and multivariable analyses of progression‐free survival in the Hellmann Cohort [14]

Parameters		*N*	Univariable analysis			Multivariable analysis		
			HR	95% CI	*P*	HR	95% CI	*P*
Age	< 60	27	0.9937	0.5538–1.783	0.9830			
	≥ 60	48						
Sex	Male	37	1.030	0.5915–1.793	0.9166			
	Female	38						
ECOG	0	30	0.6851	0.3935–1.193	0.1837			
	1	45						
Smoking	Current/former	60	0.6979	0.3304–1.474	0.2870			
	Never	15						
Histology	Squamous	16	1.177	0.5678–2.441	0.6424			
	Nonsquamous	59						
%PD‐L1 expression	> 1	43	0.7307	0.3921–1.362	0.2931			
	≤ 1	27						
*BRCA1/2*	Mut+	5	0.2663	0.1184–0.5988	0.0420	0.212	0.050–0.899	0.035
	Mut−	70						
*POLE/D1*	Mut+	4	0.4772	0.1721–1.323	0.2921			
	Mut−	71						
*PRKDC*	Mut+	6	0.2893	0.1255–0.6672	0.0650	0.240	0.058–0.998	0.050
	Mut−	69						

**Table 2:** Univariable and multivariable analyses of overall survival in the Allen Cohort [16]

Parameters		*N*	Univariable analysis			Multivariable analysis		
			HR	95% CI	*P*	HR	95% CI	*P*
Age	< 60	59	1.073	0.6971–1.652	0.7484			
	≥ 60	51						
Sex	Male	78	0.7806	0.4787–1.273	0.2924			
	Female	32						
Stage	IV	100	4.504	2.443–8.306	0.0045	4.912	1.539–15.673	0.007
	III	10						
LDH	LDH‐high	48	2.030	1.289–3.197	0.0010	2.307	1.474–3.611	0.000
	LDH‐low	58						
*BRCA1/2*	Mut+	16	0.7409	0.4184–1.312	0.3475			
	Mut−	94						
MMR	Mut+	14	0.05722	0.3249–1.008	0.1020	0.680	0.327–1.417	0.301
	Mut−	96						
*POLE/D1*	Mut+	4	0.6708	0.2566–1.753	0.4906			
	Mut−	106						
*PRKDC*	Mut+	9	0.5126	0.2715–0.9679	0.1020	0.361	0.155–0.841	0.018
	Mut−	101						

In another two cohorts [7, 15], a total of two patients (study IDs were CR4880 and PR4092, [15]) with melanoma who received anti‐CTLA4 therapy and one patient with lung adenocarcinoma (study ID was DI6359, [7]) who received anti‐PD‐1 therapy were verified to harbor *PRKDC* mutations. The numbers of nonsynonymous mutations in patients CR4880, PR4092, and DI6359 were 527, 1108, and 228, respectively. One patient achieved a complete response, and the other two patients achieved major partial responses. The overall survival for the two melanoma patients was 5.4 and 6.1 years, and the PFS for the NSCLC patient was 9.8 months (Table S7).

In our clinic, we treated a patient (NPC_Y) with stage IVA nasopharyngeal carcinoma that harbored a *PRKDC* mutation. The functional impact evaluation of the PRKDC mutation in this patient showed that it can be considered as a functional mutation (SIFT score, 0.05; PolyPhen‐2 HumVar score, 0.57). The PD‐L1 expression by IHC staining in both tumor cells and immune cells was positive (tumor proportion score > 95% and > 80%, respectively; Fig. S4). Moreover, a large number of infiltrating CD8^+ ^T cells was found in the tumor center and at the margins of all lesions (Fig. S4). The ctDNA analysis expectedly displayed a high mutation load with 26 nonsynonymous mutations (Table S8). When disease rapidly progressed with systemic metastases after first‐line therapy (left clavicle area, right lobe of liver, sternum, and T11/L4 centrum), the patient accepted nivolumab as the next‐step treatment. Treatment resulted in a complete response based on the criteria in the Response Evaluation Criteria In Solid Tumors (RECIST) 1.1, after three cycles of nivolumab. Finally, this patient had 17 months of the progression‐free survival with nivolumab (Fig. S5).

## 4 Discussion

In our study, the prevalence of *PRKDC* mutations in two large cohorts was identified in multiple solid tumor types, including common neoplasms such as gastrointestinal cancers, NSCLC, and bladder carcinoma. We are the first to comprehensively describe that *PRKDC* mutations, regardless of the status of other DDR‐related genes, and found an association with an increased TMB, an increased mRNA expression of immune‐related genes, and a superior response to ICI in pan‐cancer patients.

Double‐stranded DNA breaks are the most serious DNA lesions. The two major pathways for repair of DSB are homologous recombination (HR) and NHEJ [11, 32]. DNA‐PKcs is the key component of the NHEJ pathway involved in DSB repair [12]. *PRKDC* mutations lead to a deficiency in the DNA‐PKcs and NHEJ pathway, so DSB fail to repair and mutations tend to accumulate. Furthermore, DNA‐PKcs has been verified as essential for induction of apoptosis after massive DSB formation [33]. When *PRKDC* mutations are present, cancer cells are resistant to apoptosis and there is an increase in the accumulation of DNA damage that promotes genome instability. Moreover, such excess DNA damage may not only increase mutations due to error‐prone translesion synthesis, but also increase epigenetic alterations due to errors during DNA repair [34, 35]. In our study, we comprehensively reviewed *PRKDC* mutations and found they were significantly associated with high TMB scores in two large independent cohorts, similar to *POLE/POLD1*, MMR gene, and *BRCA1/BRCA2* mutation patients. This finding implicates *PRKDC* mutations as a valuable biomarker in clinical practice.

When performing transcriptome analysis we found that, compared to the *PRKDC* wild‐type, the patients with *PRKDC* mutations tend to have an inflamed tumor microenvironment (TME) that includes higher numbers of CD8^+ ^T cells, NK cells, Th1, and pDCs, and higher PD‐L1 expression, other immune checkpoints, and chemokine expression. The GSEA analysis also showed a remarkably upregulated expression of the IFN‐γ and IFN‐α response, and IL‐6/JAK/STAT signaling along with downregulation of TGF‐β and Wnt/β‐catenin signaling. The high TMB might be one of the reasons contributing to this inflammatory microenvironment. A previous study has shown that DNA‐PK also interacts with the transcription factor autoimmune regulator (AIRE) to promote central T‐cell tolerance [36]. Deficiency of DNA‐PK can present as an inflammatory disease with organ‐specific autoimmunity, suggesting a role of DNA‐PK in regulating autoimmune responses and maintaining AIRE‐dependent autoimmune tolerance [35]. These results support our findings that *PRKDC* mutations induced inflamed TME was caused by an increased TMB level together with impaired central immune tolerance. In the four independent cohorts, we also found that patients with *PRKDC* mutations were more likely to benefit from ICI.

However, our study has limitations. First, due to the relatively low incidence and limited data, we can only conclude that PRKDC mutation is one of the important factors which affect TMB, TME, and the prognosis of ICI therapy, but cannot confirm the extent to which PRKDC mutation sites contribute to the inflamed TME and prognosis improvements. Second, the sample size of the validation cohort was small and only melanoma and NSCLC were included, and so, the association between *PRKDC* mutation‐induced changes in the immune microenvironment and improvements of ICI treatment efficacy needs to be confirmed and verified in a larger population with multiple cancer types, such as MSS colorectal cancer and pancreatic cancer.

## 5 Conclusion

Our findings suggest that *PRKDC* mutations occur in a subset of solid tumor patients, and they often appeared to co‐exist with deficiency in some other DDR mechanism. But anyway, PRKDC mutation is still one of the important factors significantly associated with an increased TMB, an increased expression of immune‐related genes, and an improved response to ICI. Additional prospective studies are needed to validate this finding and to determine whether routine testing for this alteration is warranted. This work may have important implications for clinical practice and provide a potential predictive biomarker for guiding ICI therapy.

## Conflict of interest

The authors declare no conflict of interest.

## Author contributions

YC, CC, and YL conceived and designed the study. YG, JL, GC, LC, and XY made the collection of clinical data and sample. YG, JL, LC, and XY performed sequencing. YH, JL, LC, LP, ZG, NX, and JP analyzed the clinical data and performed statistical analysis. All authors wrote the manuscript. All authors read and approved the final manuscript.

## Supporting information


**Fig. S1.** Venn diagram of the relationship among PRKDC mutation subgroup, MSI‐H subgroup and TMB‐H subgroup. (Red: PRKDC mutation subgroup; Green: MSI‐H subgroup; Purple: TMB‐H subgroup).
**Fig. S2.** Comparison of TMB in different combinations of PRKDC mutations and MSI status groups. (ns, *P* > 0.05, *P < 0.05, **P < 0.01, ***P < 0.001, ****P < 0.0001).
**Fig. S3.** Length of transcript versus the mean number of somatic mutations in coding region for each gene in the TCGA cohorts of Bladder cancer, Colorectal cancer, Lung Adenocarcinoma and Head/Neck Squamous cell carcinoma. *R*, Pearson's correlation; curve, fitness lines. The statistics used in this analysis is Loess regression.
**Fig. S4.** The hematoxylin‐eosin (HE) staining and immunohistochemistry (IHC) staining of CD8 and PD‐L1 with the recurrent nasopharyngeal lesion of patient NPC_Y. The representative microscopic findings are shown.
**Fig. S5.** Disease course and clinical response in an advanced nasopharyngeal carcinoma patient (NPC_Y) with PRKDC mutation treated with Nivolumab‐based multi‐combination strategies.
**Table S1.** The cancer types and number of samples included in Geneplus pan‐cancer cohort.
**Table S2.** The logistic regression of factors influencing TMB‐high in TCGA top 10 cancers.
**Table S3.** The logistic regression of factors influencing TMB‐high in the combined four cohorts which with MSI/MSS data.
**Table S4.** GSEA between PRKDC mutation group and non‐mutation group with the Hallmark gene set in TCGA top 10 cancers dataset.
**Table S5.** Gene list of immune‐related gene set.
**Table S6.** Immune‐related gene set mRNA expression analysis.
**Table S7.** The clinical data of three patients with PRKDC mutation from two clinical cohorts treated with ICIs.
**Table S8.** The mutation list of an advanced nasopharyngeal carcinoma patient (NPC_Y) by ctDNA analysis.
**Appendix S1.** Case report.
**Appendix S2.** Immunohistochemical staining and analysis for CD8, PD‐L1.

## Data Availability

The TCGA datasets analyzed in the study are available at the cBioPortal (www.cbioportal.org/). Relevant data in this study were provided in the supplementary information. Other data could be obtained from the corresponding authors of this study.

## Data accessibility

The TCGA datasets analyzed in the study are available at the cBioPortal (www.cbioportal.org/). Relevant data in this study were provided in the supplementary information. Other data could be obtained from the corresponding authors of this study.
