# Supplementary material for: Prevalence of PRKDC mutations and association with response to immune checkpoint inhibitors in solid tumors
Source: Mol Oncol. 2020 Jun 30;14(9):2096–110. doi: 10.1002/1878-0261.12739 (PMC7463346; doi:10.1002/1878-0261.12739)
Supplement: Supplementary file 1 — Fig. S1. Venn diagram of the relationship among PRKDC mutation subgroup, MSI‐H subgroup and TMB‐H subgroup. (Red: PRKDC mutation subgroup; Green: MSI‐H subgroup; Purple: TMB‐H subgroup). Fig. S2. Comparison of TMB in different combinations of PRKDC mutations and MSI status groups. (ns, P > 0.05, *P < 0.05, **P < 0.01, ***P < 0.001, ****P < 0.0001). Fig. S3. Length of transcript versus the mean number of somatic mutations in coding region for each gene in the TCGA cohorts of Bladder cancer, Colorectal cancer, Lung Adenocarcinoma and Head/Neck Squamous cell carcinoma. R, Pearson's correlation; curve, fitness lines. The statistics used in this analysis is Loess regression. Fig. S4. The hematoxylin‐eosin (HE) staining and immunohistochemistry (IHC) staining of CD8 and PD‐L1 with the recurrent nasopharyngeal lesion of patient NPC_Y. The representative microscopic findings are shown. Fig. S5. Disease course and clinical response in an advanced nasopharyngeal carcinoma patient (NPC_Y) with PRKDC mutation treated with Nivolumab‐based multi‐combination strategies. Table S1. The cancer types and number of samples included in Geneplus pan‐cancer cohort. Table S2. The logistic regression of factors influencing TMB‐high in TCGA top 10 cancers. Table S3. The logistic regression of factors influencing TMB‐high in the combined four cohorts which with MSI/MSS data. Table S4. GSEA between PRKDC mutation group and non‐mutation group with the Hallmark gene set in TCGA top 10 cancers dataset. Table S5. Gene list of immune‐related gene set. Table S6. Immune‐related gene set mRNA expression analysis. Table S7. The clinical data of three patients with PRKDC mutation from two clinical cohorts treated with ICIs. Table S8. The mutation list of an advanced nasopharyngeal carcinoma patient (NPC_Y) by ctDNA analysis. Appendix S1. Case report. Appendix S2. Immunohistochemical staining and analysis for CD8, PD‐L1. [file MOL2-14-2096-s001.docx]

**Supplementary Materials**

**Appendix S1.** Case report.

**Appendix S2.** Immunohistochemical staining and analysis for CD8, PD-L1.

**Supplementary Table S1.** The cancer types and number of samples included in Geneplus pan-cancer cohort.

**Supplementary Table S2.** The logistic regression of factors influencing TMB-high in TCGA top 10 cancers.

**Supplementary Table S3.** The logistic regression of factors influencing TMB-high in the combined four cohorts which with MSI/MSS data.

**Supplementary Table S4.** Gene set enrichment analysis (GSEA) between PRKDC mutation group and non-mutation group with the Hallmark gene set in TCGA top 10 cancers dataset.

**Supplementary Table S5.** Gene list of immune-related gene set.

**Supplementary Table S6.** Immune-related gene set mRNA expression analysis.

**Supplementary Table S7.** The clinical data of three patients with PRKDC mutation from two clinical cohorts treated with ICIs.

**Supplementary Table S8.** The mutation list of an advanced nasopharyngeal carcinoma patient (NPC_Y) by ctDNA analysis.

**Supplementary Figure S1.** Venn diagram of the relationship among PRKDC mutation subgroup, MSI-H subgroup and TMB-H subgroup. (Red: PRKDC mutation subgroup; Green: MSI-H subgroup; Purple: TMB-H subgroup.)

**Supplementary Figure S2.** Comparison of TMB in different combinations of PRKDC mutations and MSI status groups. (ns, P > 0.05, *, P < 0.05, **, P < 0.01, ***, P < 0.001, ****P <0.0001)

**Supplementary** **Figure S3.** Length of transcript versus the mean number of somatic mutations in coding region for each gene in the TCGA cohorts of Bladder cancer, Colorectal cancer, Lung Adenocarcinoma and Head/Neck Squamous cell carcinoma. R, Pearson’s correlation; curve, fitness lines. The statistics used in this analysis is Loess regression.

**Supplementary Figure S4.** The hematoxylin-eosin (HE) staining and immunohistochemistry (IHC) staining of CD8 and PD-L1 with the recurrent nasopharyngeal lesion of patient NPC_Y. The representative microscopic findings are shown.

**Supplementary Figure S5.** Disease course and clinical response in an advanced nasopharyngeal carcinoma patient (NPC_Y) with PRKDC mutation treated with Nivolumab-based multi-combination strategies.

**Supplementary Methods**

**Case report**

A 47-year-old man was diagnosed with a stage IVA Nasopharyngeal non-keratinized undifferentiated carcinoma (NPC_Y). He had no specific medical history and familial history of cancer. After treatment of first-line 2 cycles Paclitaxel plus Lobaplatin neoadjuvant chemotherapy followed by concurrent chemoradiotherapy (IMRT DT7000cGy/35F/7W+ 2 cycles Lobaplatin) from May 24, 2016 to August 9, 2016, the disease rapidly progressed three months later. On November 15, 2016, PET-CT confirmed nasopharyngeal tumor recurrence accompanied by systemic metastases (left clavicle area, right lobe of liver, sternum and T11/L4 centrum). IHC indicated that a strong expression of PD-L1 both in tumoral cells (TPS≥90%) and in infiltrating immune cells, a large amount of CD8+ T cell infiltration, both in primary lesion and left cervical metastatic lymph node. Then he received Nivolumab (3mg/kg q2w) for 6 cycles from November 24, 2016 to May 12, 2017. At the time of he finished the third cycle nivolumab treatment (On January 29), PET-CT showed the recurrent and metastatic lesions complete response (CR), according to RECIST 1.1. After six cycles, the patient stopped the nivolumab treatment. On December 14, 2017, the patient’s abdominal CT scan showed newly enlarged Hilar lymph nodes, the tumor progression again, the progression-free survival was 13 months (PFS1). And at this time, somatic mutation in PRKDC was detected in the patient’s blood ctDNA(variant allele frequency was 1.97%). Then a total 8 cycles nivolumab (3mg/kg q2w) were given discontinuously from January 14, 2018 to February 20, 2019, and once again, it had indicated significant clinical benefit. After the fourth cycle nivolumab, PEC-CT showed complete response (CR), and until May 28, 2019, the imaging data still showed complete remission, the progression-free survival is 17 months (PFS2).

**Immunohistochemical staining and analysis for CD8, PD-L1**

Serial FFPE tissue sections of 4μm thickness were assayed for CD8, PD-L1. The IHC assay was performed using the 2-step EnVision IHC procedure (Dako, Glostrup, Denmark). All staining was performed on a Dako Autostainer (link 48). Each section was dewaxed in xylene, rehydrated in gradient ethanol solutions, and heated with EDTA (pH 9.0) in an autoclave for antigen retrieval. Endogenous peroxidase activity was blocked with hydrogen peroxide. Sections were incubated with primary antibodies against CD8 (rabbit monoclonal primary antibody, SP16, Biocare Medical), PD-L1 (rabbit monoclonal primary antibody, 28-8, Abcam) at 4°C overnight, followed by incubation with secondary antibody for 30 minutes. Immunostaining was visualized with diaminobenzidine and counterstained with hematoxylin. PD-L1 expression was assessed by tumor proportion score (TPS), which was defined as the percentage of tumor cells with membranous PD-L1 staining[[1](#_ENREF_1)].

**Supplementary Table S1.** The cancer types and number of samples included in Geneplus pan-cancer cohort.

| **Cancer types** | **The number of samples** |
| --- | --- |
| Melanoma | 17 |
| Small cell lung cancer | 55 |
| Cervical cancer | 30 |
| Bladder urothelial carcinoma | 86 |
| Colorectal adenocarcinoma | 854 |
| Head and Neck cancer | 100 |
| Breast cancer | 272 |
| Non-small cell lung cancer | 2463 |
| Total | 3877 |

**Supplementary Table S2.** The logistic regression of factors influencing TMB-high in TCGA top 10 cancers.

| **Risk factors** | **Regression Coefficient** | **Standard Error** | **P** | **OR** | **95%CI** |
| --- | --- | --- | --- | --- | --- |
| Age | 0.010 | 0.003 | 0.002 | 1.010 | 1.004-1.016 |
| PRKDC mutation | 1.376 | 0.141 | 0.000 | 3.958 | 3.003-5.216 |
| BRCA1/2 mutation | 1.455 | 0.127 | 0.000 | 4.283 | 3.342-5.490 |
| POLE/D1 mutation | 1.465 | 0.148 | 0.000 | 4.326 | 3.238-5.780 |
| MMR genes mutation | 1.484 | 0.150 | 0.000 | 4.413 | 3.291-5.916 |

**Supplementary Table S3.** The logistic regression of factors influencing TMB-high in the combined four cohorts which with MSI/MSS data.

| **Risk factors** | **Regression Coefficient** | **Standard Error** | **P** | **OR** | **95%CI** |
| --- | --- | --- | --- | --- | --- |
| PRKDC mutation | 2.967 | 0.642 | 0.000 | 19.428 | 5.525-68.317 |
| MSI-H | 3.564 | 0.279 | 0.000 | 35.318 | 20.447-61.005 |
| BRCA1/2 mutation | 1.717 | 0.469 | 0.000 | 5.567 | 2.221-13.951 |
| MMR genes mutation | 1.017 | 0.508 | 0.045 | 2.764 | 1.022-7.473 |
| POLE/D1  mutation | 1.800 | 0.458 | 0.000 | 6.049 | 2.467-14.831 |

**Supplementary Table S4.** Gene set enrichment analysis (GSEA) between PRKDC mutation group and non-mutation group with the Hallmark gene set in TCGA top 10 cancers dataset.

| **Up-regulated pathways** | | | | | | |
| --- | --- | --- | --- | --- | --- | --- |
| **Name** | **ES** | **NES** | **NOM p-val** | **FDR q-val** | **FWER p-val** | **Top-scoring genes** |
| HALLMARK_E2F_TARGETS | 0.619038 | 3.200738 | 0 | 0 | 0 | *CDCA8, DEPDC1, BARD1, LMNB1, DCK, SPC25, DCLRE1B, SLBP, PAICS, MAD2L1* |
| HALLMARK_G2M_CHECKPOINT | 0.58132 | 2.989083 | 0 | 0 | 0 | *STIL, PBK, BUB1, CCNA2, SAP30, NSD2, BARD1, LMNB1, KIF11, FBXO5* |
| HALLMARK_INTERFERON_GAMMA_RESPONSE | 0.476733 | 2.448866 | 0 | 0 | 0 | *RIPK2, IRF1, ISOC1, CASP3, GBP4, APOL6, GCH1, NLRC5, BATF2, SECTM1* |
| HALLMARK_INTERFERON_ALPHA_RESPONSE | 0.50786 | 2.348955 | 0 | 0 | 0 | *RIPK2, IRF1, GBP4, BATF2, LAP3, NUB1, NMI, TAP1, GBP2, NCOA7* |
| HALLMARK_MYC_TARGETS_V1 | 0.423836 | 2.188747 | 0 | 0 | 0 | *C1QBP, CCNA2, PRDX3, TYMS, DHX15, HNRNPR, MAD2L1, BUB3, IARS, GNL3* |
| HALLMARK_MTORC1_SIGNALING | 0.39987 | 2.066417 | 0 | 0 | 0 | *BUB1, STARD4, ELOVL6, DHFR, HSP90B1, RRM2, MTHFD2, EEF1E1, INSIG1, OLR3G* |
| HALLMARK_ALLOGRAFT_REJECTION | 0.391989 | 2.0435 | 0 | 0 | 0 | *RIPK2, LYN, KLRD1, CCL4, TAP1, IFNG, GBP2, NCR1, FASLG, HLA-DMA* |
| HALLMARK_MITOTIC_SPINDLE | 0.388658 | 2.000778 | 0 | 2.23E-04 | 0.001 | *BUB1, RHOF, LMNB1, KIF11, FBXO5, NEK2, KIF15, CENPE, KIF2C, GEMIN4* |
| HALLMARK_SPERMATOGENESIS | 0.398829 | 1.862598 | 0 | 0.001148 | 0.007 | *ACRBP, BUB1, LPIN1, PIAS2, GFI1, NEK2, EZH2, KIF2C, NCAPH, SLC2A5* |
| HALLMARK_MYC_TARGETS_V2 | 0.44589 | 1.860773 | 0 | 0.001033 | 0.007 | *PLK4 , SUPV3L1 , GNL3 , NOLC1 , SORD , MCM4 , UNG , PLK1 , SLC29A2 , NOP2* |
| HALLMARK_UNFOLDED_PROTEIN_RESPONSE | 0.362436 | 1.709411 | 0 | 0.003111 | 0.021 | *ALDH18A1, HSP90B1, MTHFD2, GEMIN4, SSR1, HSPA5, IARS, NOLC1, EXOSC9, HSPA9* |
| HALLMARK_OXIDATIVE_PHOSPHORYLATION | 0.332411 | 1.70486 | 0 | 0.002852 | 0.021 | *PRDX3, SDHB, ATP5A1, ECHS1, CASP7, NDUFA9, SDHA, DLAT, COX10, COX15* |
| HALLMARK_PROTEIN_SECRETION | 0.331325 | 1.524556 | 0.005666 | 0.013933 | 0.12 | *SEC24D, LMAN1, TMED2, ANP32E, ARCN1, VPS4B, AP2B1, GOLGA4, NAPG, KRT18* |
| HALLMARK_COMPLEMENT | 0.289254 | 1.483264 | 0.006079 | 0.017913 | 0.162 | *XPNPEP1, IRF1, LYN, CASP3, PCSK9, LAP3, CASP10, CASP7, CALM3, HSPA5* |
| HALLMARK_IL6_JAK_STAT3_SIGNALING | 0.309333 | 1.415464 | 0.02981 | 0.031369 | 0.283 | *IRF1 , CXCL3 , IL15RA , IL7 , PTPN11 , GRB2 , CXCL9 , FAS , MYD88 , IL2RA* |
| HALLMARK_GLYCOLYSIS | 0.264963 | 1.383487 | 0.006329 | 0.04025 | 0.369 | *ME2 , PKP2 , DEPDC1 , SAP30 , FUT8 , SLC35A3 , PAXIP1 , GFPT1 , HSPA5 , P4HA1* |
| **Down-regulated pathway** | | | | | | |
| **Name** | **ES** | **NES** | **NOM p-val** | **FDR q-val** | **FWER p-val** | **Top-scoring genes** |
| HALLMARK_EPITHELIAL_MESENCHYMAL_TRANSITION | -0.408154 | -1.955634 | 0 | 6.76E-04 | 0.001 | *SGCB , IL32 , FAS , VEGFA , ANPEP , FMOD , CXCL1 , PLAUR , CXCL8 , LOX…* |
| HALLMARK_COAGULATION | -0.414253 | -1.870583 | 0 | 0.00107 | 0.003 | *ARF4 , ANG , PREP , GDA , MMP15 , CAPN5 , MST1 , C8G , ADAM9 , PLEK …* |
| HALLMARK_KRAS_SIGNALING_DN | -0.381975 | -1.78586 | 0 | 0.002498 | 0.01 | *BARD1 , IFNG , HTR1D , MAST3 , GPR3 , YBX2 , SLC25A23 , NR4A2 , NPHS1 , OXT…* |
| HALLMARK_MYOGENESIS | -0.372101 | -1.756585 | 0 | 0.002664 | 0.014 | *LPIN1 , TPM3 , CRAT , IFRD1 , AGL , FABP3 , PDE4DIP , FDPS , ERBB3 , ACSL1…* |
| HALLMARK_APICAL_JUNCTION | -0.347823 | -1.686475 | 0 | 0.004698 | 0.03 | *RHOF, CLDN7, NECTIN3, INSIG1, YWHAH, CRAT , TIAL1 , EPB41L2 , PIK3CB , ADAM9…* |
| HALLMARK_HEDGEHOG_SIGNALING | -0.468602 | -1.667926 | 0.008389 | 0.005366 | 0.041 | *DDAH1, INSIG1, AGGF1, BCKDHB, SFMBT1, ADD3, PAPD7, GCNT1, CDC42BPA, LDLR…* |
| HALLMARK_WNT_BETA_CATENIN_SIGNALING | -0.441004 | -1.610866 | 0.008518 | 0.007691 | 0.069 | *DDAH1, INSIG1, AGGF1, BCKDHB, SFMBT1, ADD3, PAPD7, GCNT1, CDC42BPA, LDLR…* |
| HALLMARK_UV_RESPONSE_DN | -0.347453 | -1.591471 | 0 | 0.007952 | 0.081 | *DDAH1, INSIG1, AGGF1, BCKDHB, SFMBT1, ADD3, PAPD7, GCNT1, CDC42BPA, LDLR…* |
| HALLMARK_TGF_BETA_SIGNALING | -0.377115 | -1.471187 | 0.025518 | 0.031023 | 0.306 | *RHOA, SPTBN1, TGIF1, SMURF2, UBE2D3, TRIM33, APC, PPM1A, SMAD6, SMAD1* |

**Supplementary Table S5.** Gene list of immune-related gene set.

| **Gene set** | **Gene** |
| --- | --- |
| CD8 T cells | *CD8A* |
| Chemokines | *CCL18, CCL19, CCL21, CCL2, CCL3, CCL4, CCL5, CXCL10, CXCL11, CXCL9* |
| Checkpoint | *PDCD1, CD274, CTLA4, LAG3* |
| CTL (cytotoxic lymphocyte) | *GZMA, GZMB, CD8A, PRF1* |
| NK cells | *KLRC1, KLRF1, NCR1, NCR2* |
| Th1 | *IFNG, TBX21* |
| pDCs | *LILRA4, CLEC4C, PLD4, PHEX, IL3RA, IRF8, IRF7, GZMB, CXCR3* |
| Stress | *MICB, RAET1G, ULBP1, ULBP2, ULBP3, RAET1E* |
| Anti-inflammatory cytokines | *EBI3, TGFB1, TGFB2, IL10* |
| Macrophages | *FACA1, MMP9, LGMN, HS3ST2, TM4SF19, GPNMB, C11orf45, CD68, CYBB* |
| Antigen presentation machinery | *PSMB8, PSMB10, HSPA5, CANX, CALR, PDIA3, TAPBP, HLA-B, HLA-C, B2M, IFNG, IFNGR1, IFNGR2, JAK1, JAK2, STAT1* |
| CD4 Treg | *FOXP3* |
| CD4 T cells | *CD4* |
| Neutrophils | *KDM6B, HSD17B11, EVI2B, MNDA, MEGF9, SELL, NLRP12, PADI4, TRANK1, VNN3* |

**Supplementary Table S6.** Immune-related gene set mRNA expression analysis

| Gene set | Mean rank | | *P* value |
| --- | --- | --- | --- |
|  | PRKDC mut^+^ group | PRKDC mut^-^ group |  |
| CD8 T cells | 1939.96 | 1756.21 | .004 |
| Chemokines | 1937.85 | 1756.40 | .004 |
| Checkpoint | 1484.56 | 1318.46 | .003 |
| CTL (cytotoxic lymphocyte) | 1945.76 | 1755.70 | .003 |
| NK cells | 1937.41 | 1756.43 | .004 |
| Th1 | 1970.11 | 1753.57 | .001 |
| pDCs | 1890.71 | 1760.52 | .039 |
| Stress | 1893.73 | 1760.26 | .035 |
| Anti-inflammatory cytokines | 1715.54 | 1775.85 | .340 |
| Macrophages | 1706.21 | 1776.67 | .265 |
| Antigen presentation machinery | 1847.49 | 1764.30 | .188 |
| CD4 Treg | 1793.58 | 1769.02 | .697 |
| CD4 T cells | 1803.79 | 1768.13 | .565 |
| Neutrophils | 1879.33 | 1761.52 | .062 |

**Supplementary Table S7.** The clinical data of three patients with PRKDC mutation from two clinical cohorts treated with ICIs.

| Snyder cohort[[2](#_ENREF_2)] | | | | | | | | | | | | | |
| --- | --- | --- | --- | --- | --- | --- | --- | --- | --- | --- | --- | --- | --- |
| Study ID | Age | Gender | M stage | Ipilimumab dosing(mg/kg×#) | | Response duration(weeks) | | | | OS (year) | TMB**^*^** | | |
| CR4880 | 63 | M | M1b | 10×16 | | 59 | | | | 5.4 | 527 | | |
| PR4092 | 57 | F | M1a | 10×4 | | 315 | | | | 6.1 | 1108 | | |
| Rizvi cohort[[3](#_ENREF_3)] | | | | | | | | | | | | | |
| Study ID | Age | Gender | Histology | Pembrolizumab dosing(mg/kg) | PFS (mos) | | Event | Response | Durable clinical benefit | | | TMB^*^ | TNB^**^ |
| DI6359 | 61 | F | Adeno | 10 | 9.8 | | 0 | PR | DCB | | | 228 | 215 |

*TMB was defined as the number of nonsynonymous mutations.

**TNB was defined as the number of predicted neoantigen

**Supplementary Table S8.** The mutation list of an advanced nasopharyngeal carcinoma patient (NPC_Y) by ctDNA analysis.

| **Gene Symbol** | **cHGVS** | **pHGVS** | **Function** | **AF (%)** |
| --- | --- | --- | --- | --- |
| *STAG2* | c.646C>T | p.R216* | Nonsense | 6.6667 |
| *TP53* | c.687T>A | p.C229* | Nonsense | 5.2332 |
| *JAK2* | c.2643A[6>5] | p.K883Sfs*10 | Frameshift | 4.5191 |
| *RUNX1* | c.14G>A | p.S5N | Missense | 4.417 |
| *ELAC2* | c.2131G>A | p.V711M | Missense | 3.5454 |
| *ZMAT3* | c.377C>T | p.P126L | Missense | 3.2258 |
| *MTOR* | c.518G>A | p.R173H | Missense | 3.0939 |
| *CDK12* | c.2735G>A | p.R912H | Missense | 2.8736 |
| *DNMT3A* | c.1601A>G | p.Q534R | Missense | 2.8302 |
| *DDR2* | c.833T>C | p.I278T | Missense | 2.7586 |
| *RAF1* | c.769_771delTCG | p.S257del | cds-del | 2.4279 |
| *G3BP2* | c.1275G>T | p.R425S | Missense | 2.4138 |
| *FLT4* | c.139C>A | p.L47M | Missense | 2.3553 |
| *CTCF* | c.1480C>T | p.R494C | Missense | 2.2879 |
| *EGFR* | c.1169A>G | p.Q390R | Missense | 2.2727 |
| *STAT1* | c.1385G>A | p.S462N | Missense | 2.1898 |
| *CREBBP* | c.1802G>A | p.R601Q | Missense | 2.0994 |
| *PRKDC* | c.5635G>A | p.V1879M | Missense | 1.9763 |
| *ASXL1* | c.794G>A | p.R265H | Missense | 1.9459 |
| *NRAS* | c.436G>A | p.A146T | Missense | 1.9355 |
| *EPHB1* | c.2164G>A | p.V722M | Missense | 1.875 |
| *THRAP3* | c.1799G>T | p.S600I | Missense | 1.6888 |
| *SEMA3A* | c.2197C>T | p.R733C | Missense | 1.5299 |
| *FOXA1* | c.1216_1217delAT | p.M406Vfs*8 | Frameshift | 1.3853 |
| *IMPG1* | c.196C>T | p.H66Y | Missense | 1.3158 |
| *ARID1B* | c.5695G>A | p.E1899K | Missense | 0.5613 |

**Supplementary Figure S1.** Venn diagram of the relationship among PRKDC mutation subgroup, MSI-H subgroup and TMB-H subgroup. (Red: PRKDC mutation subgroup; Green: MSI-H subgroup; Purple: TMB-H subgroup.)

**Supplementary Figure S2.** Comparison of TMB in different combinations of PRKDC mutations and MSI status groups. (ns, P > 0.05, *, P < 0.05, **, P < 0.01, ***, P < 0.001, ****P <0.0001)


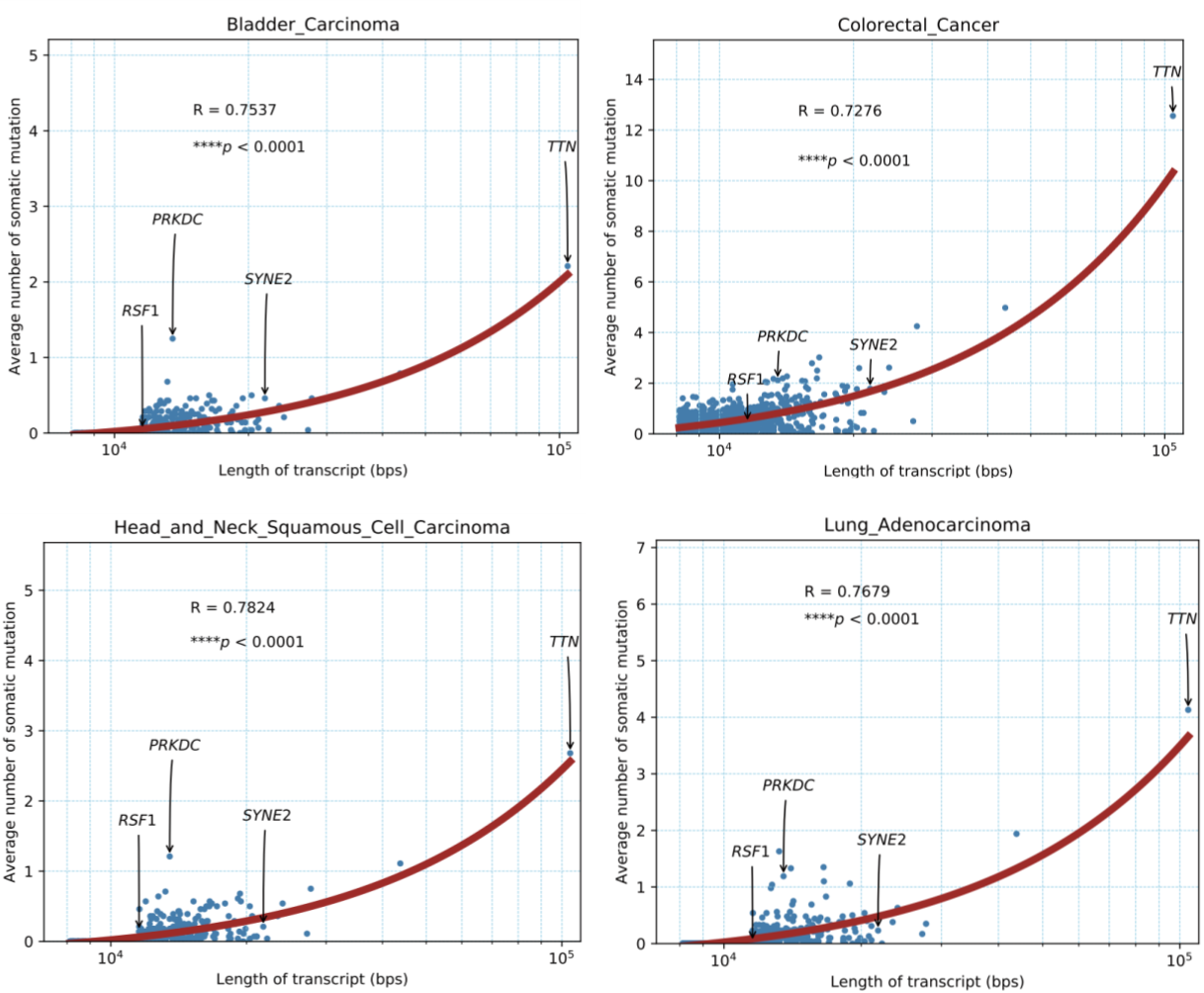


**Supplementary Figure S3.** Length of transcript versus the mean number of somatic mutations in coding region for each gene in the TCGA cohorts of Bladder cancer, Colorectal cancer, Lung Adenocarcinoma and Head/Neck Squamous cell carcinoma. R, Pearson’s correlation; curve, fitness lines. The statistics used in this analysis is Loess regression.


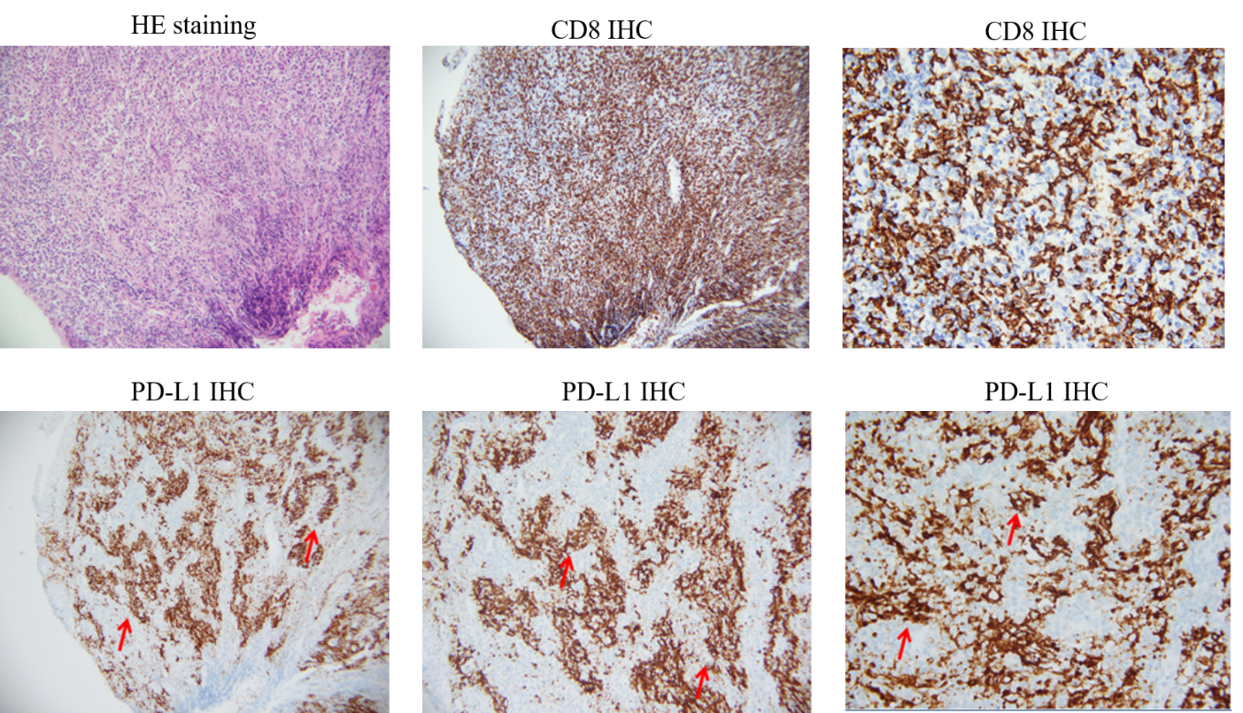
**Supplementary Figure S4.** The hematoxylin-eosin (HE) staining and immunohistochemistry (IHC) staining of CD8 and PD-L1 with the recurrent nasopharyngeal lesion of patient NPC_Y. The representative microscopic findings are shown.


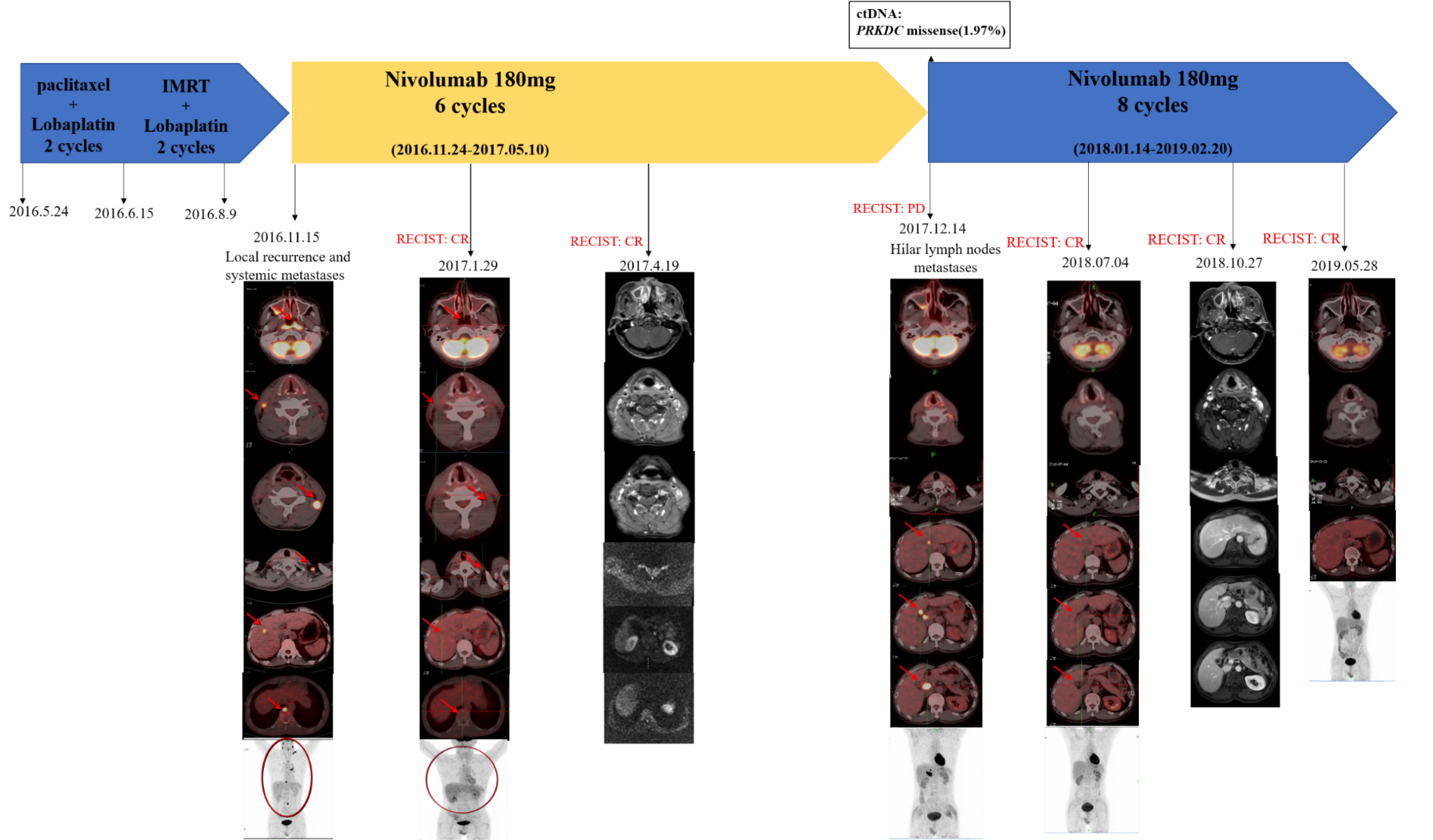


**Supplementary Figure S5.** **Disease course and clinical response in an advanced nasopharyngeal carcinoma patient (NPC_Y) with *PRKDC* mutation treated with Nivolumab-based multi-combination strategies.** Disease course and tumor progression baseline, follow-up positron emission tomography/computed tomography (PET/CT) and magnetic resonance images (MRI) in NPC_Y. Arrows show locations of lesions. Abbreviations: IMRT, Intensity modulated radiotherapy; RECIST, Response Evaluation Criteria in Solid Tumor.

**References**

1. Roach C, Zhang N, Corigliano E, Jansson M, Toland G, Ponto G, Dolled-Filhart M, Emancipator K, Stanforth D, Kulangara K: **Development of a Companion Diagnostic PD-L1 Immunohistochemistry Assay for Pembrolizumab Therapy in Non-Small-cell Lung Cancer.** *Appl Immunohistochem Mol Morphol* 2016, **24:**392-397.

2. Snyder A, Makarov V, Merghoub T, Yuan J, Zaretsky JM, Desrichard A, Walsh LA, Postow MA, Wong P, Ho TS, et al: **Genetic basis for clinical response to CTLA-4 blockade in melanoma.** *N Engl J Med* 2014, **371:**2189-2199.

3. Rizvi NA, Hellmann MD, Snyder A, Kvistborg P, Makarov V, Havel JJ, Lee W, Yuan J, Wong P, Ho TS, et al: **Cancer immunology. Mutational landscape determines sensitivity to PD-1 blockade in non-small cell lung cancer.** *Science* 2015, **348:**124-128.
